# Kinematic and Temporal Differences Between World-Class Men's and Women's Hurdling Techniques

**DOI:** 10.3389/fspor.2022.873547

**Published:** 2022-04-29

**Authors:** Athanassios Bissas, Giorgos P. Paradisis, Brian Hanley, Stéphane Merlino, Josh Walker

**Affiliations:** ^1^Carnegie School of Sport, Leeds Beckett University, Leeds, United Kingdom; ^2^Athletics Biomechanics, Leeds, United Kingdom; ^3^School of Sport and Exercise, University of Gloucestershire, Gloucester, United Kingdom; ^4^School of Physical Education and Sport Science, National and Kapodistrian University of Athens, Athens, Greece; ^5^International Relations and Development Department, World Athletics, Monte Carlo, Monaco

**Keywords:** hurdles, athletics, sprinting, world-class, statistical parametric mapping

## Abstract

This study aimed to compare joint kinematics and center of mass parameters throughout hurdle clearance between world-class men and women sprint hurdlers, who were competing in a World Championships final. This was the first study to present time-series kinematic data around hurdle clearance, and given the technical ability of the athletes analyzed, it can be used as a template when analyzing the technique of other athletes in similar competitions and training. Video data were collected of the 16 finalists at the 2017 IAAF World Championships using four high-speed cameras (150 Hz). Video files were continuously digitized manually from touchdown before hurdle clearance to toe-off after landing around the sixth hurdle for men and the fifth hurdle for women, and sex-based comparisons were made at key discrete time points using independent *t*-tests, and throughout the entire hurdle phase using statistical parametric mapping. When calculated relative to hurdle height, the women's center of mass height was significantly greater than the men's throughout the full analyzed sequence (*p* < 0.001). Men also displayed more hip flexion in the lead leg at take-off before hurdle clearance (*p* = 0.029) as well as a more extended knee joint at intervals during flight and upon landing (*p* ≤ 0.037). Women completed the hurdle phase in a significantly shorter time than men (~11% difference, *p* < 0.001). Finally, women seemed to be more efficient by maintaining and even exceeding their entry velocity for the first 40% of the hurdle phase. These results show a lower technical demand for the women to successfully negotiate hurdle clearance, thus providing further evidence to support the argument that the women's hurdle height is too low for their performance capabilities and should be raised in senior competition.

## Introduction

The men's and women's high hurdles are short-distance track and field athletics events included in all outdoor major championship events. The men's event covers 110 m, with a total of 10 × 1.067-m high hurdles located every 9.14 m from a 13.72-m run-in, leaving a 14.02-m run-out (World Athletics, 2019). The women's event is over 100 m, with 10 × 0.838-m high hurdles located every 8.50 m from a 13.00-m run-in, leaving a 10.50-m run-out (World Athletics, 2019). The current world record (WR) for the men's event is 12.80 s, set in 2012 (World Athletics, 2021), and for the women's event is 12.20 s, set in 2016 (World Athletics, 2021). These times correspond to a mean race speed of 8.59 and 8.20 m/s for the men's and women's WRs, respectively. Despite men displaying a higher absolute speed compared with women over what is a slightly longer distance, these mean speeds correspond to 82% and 86% of the respective 100 m flat sprint events for men and women (using WR times of 9.58 and 10.49 s; World Athletics, 2021). Therefore, clearing hurdles obviously limits the maximum running speed achieved by athletes (McDonald and Dapena, 1991; Graubner and Nixdorf, 2011). However, it is also plausible to suggest that the different task constraints between the men's and women's hurdles events (e.g., height of hurdle, distance between hurdles) might limit performance differently between sexes.

The discrepancy in hurdle heights, and its impact on hurdle clearance technique, has been recently studied in competition during the World Championships finals (Hanley et al., 2021), showing that the height of the whole-body center of mass (CM) was comparable between sexes at key events around hurdle take-off and landing, when displayed relative to athlete stature. However, when CM height was displayed relative to hurdle height, the women showed higher values at initial contact and take-off before hurdle clearance, and initial contact and toe-off after hurdle clearance (Hanley et al., 2021). Women were also, in relative terms, able to take-off farther from the hurdle, and achieve an absolute and relative shorter landing distance post-hurdle, meaning step length could be increased in the recovery (Hanley et al., 2021). It was suggested that these differences provide the women with a mechanical advantage over their counterparts in the men's event, making the task of hurdle clearance, and thus the event, less demanding for them. A need for a revision of the current hurdle heights is not a novel idea. Etcheverry (1993) explained that regulations in women's hurdles (race distance, distance between hurdles, height of hurdles) were modified in 1969. This was predominantly because of increases in popularity of the event and the performance of the athletes. However, these regulations have remained unchanged since, despite other changes in women's athletics (e.g., the introduction of the pole vault, steeplechase and triple jump at major championships). Etcheverry (1993) therefore recommended an increase in women's hurdle heights to 0.914 m, which would be comparable to the men's in terms of hurdle height relative to the average athlete's stature and would of course increase the technical and mechanical demand of hurdling for women. However, before recommendations can be made regarding the changes to race regulations, a deeper exploration into the kinematic characteristics of hurdling is required to fully understand the impact of the lesser relative hurdle height in women.

Statistical techniques such as statistical parametric mapping (SPM) (Pataky, 2010) have been used to analyze *n*-dimensional biomechanical data including kinetic and kinematic information (e.g., Pataky et al., 2013) or muscle activity data (e.g., Robinson et al., 2015). These methods of analyses have increased in popularity in recent years because of the development of publicly available software packages and allow researchers to explore biomechanical variables throughout the duration of a movement or phase, such as the stance phase of running. Various studies have used these techniques in the analysis of track and field sprint events (e.g., Colyer et al., 2018, 2019; Nagahara et al., 2021). Nagahara et al. (2021) used SPM to investigate the relationships between ground reaction forces during hurdling and four step cycle time (preparatory step to preparatory step time) in a group of well-trained male hurdlers (110 m hurdles personal best: 14.52 ± 0.60 s). It was found that brief phases throughout the hurdle step, landing step, and recovery step displayed significant relationships with cycle time. These findings have improved our understanding of performance requirements for effective hurdling.

Nonetheless, there is a lack of information in the literature about the joint and CM kinematic characteristics of hurdle clearance, especially in the one-dimensional time-series. Furthermore, there is a lack of information available on world-class athletes in the hurdle events, especially during competition. It should be appreciated that the collection of ground reaction forces is usually impossible within competition, because of the need for a regulated running surface and the fact they usually take place in outdoor stadiums. Therefore, in-competition data are usually restricted to spatiotemporal and kinematic information obtained from high-speed videography (e.g., Pollitt et al., 2018a,b; Hanley et al., 2021). In addition, the reference to a group of “world-class” athletes has recently been discussed (McKay et al., 2021), where it was highlighted that such high-standard athletes probably make-up <0.00006% of the global population. In track and field events, world-class athletes are Olympic or World Championship finalists in their event and typically achieve performances within 2% of the current world leading or WR performance. As such, these athletes race together on the world stage every two years only (at least), making them an incredibly difficult population to analyze in a single study during competition. Considering the limitations of the available research, the aim of the current study was to provide time-series kinematic information of the hurdle clearance during a World Championship final in world-class men and women hurdlers. The study also aimed to compare kinematic characteristics between men and women to more comprehensively explore the potential mechanical advantage offered to women due to lower hurdle heights. These results build on the spatiotemporal and kinematic characteristics presented in Hanley et al. (2021) by showing how the differing hurdle heights affect joint kinematics throughout the entire hurdle phase, not just at key discrete time points.

## Materials and Methods

### Research Approval

All data were collected as part of the London 2017 World Championships Biomechanics Research Project (Pollitt et al., 2018a,b). Use of the data for this study was approved by the IAAF (since renamed World Athletics), who own and control the data, and locally the study was reviewed and approved by Carnegie School of Sport Research Ethics Committee. The participants provided their written informed consent to participate in this study. The study was conducted in accordance with the recognized ethical standards of the Declaration of Helsinki.

### Participants

The eight finalists from the men's 110 m hurdles (age: 27 ± 3 years; stature: 1.87 ± 0.05 m) and the eight finalists from the women's 100 m hurdles (age: 27 ± 3 year; stature: 1.68 ± 0.04 m) were analyzed in this study. The athletes' dates of birth and finishing times were obtained from the open-access World Athletics website (World Athletics, 2021) for competitors in both races. Athletes' statures were obtained from Matthews (2017). Athlete personal bests in their respective events were 13.00 ± 0.14 s for the men (range: 12.80 s [WR] to 13.21 s), and 12.49 ± 0.20 s for the women (range: 12.20 s [WR] to 12.78 s) (World Athletics, 2021).

### Data Collection

**Figure 1:**
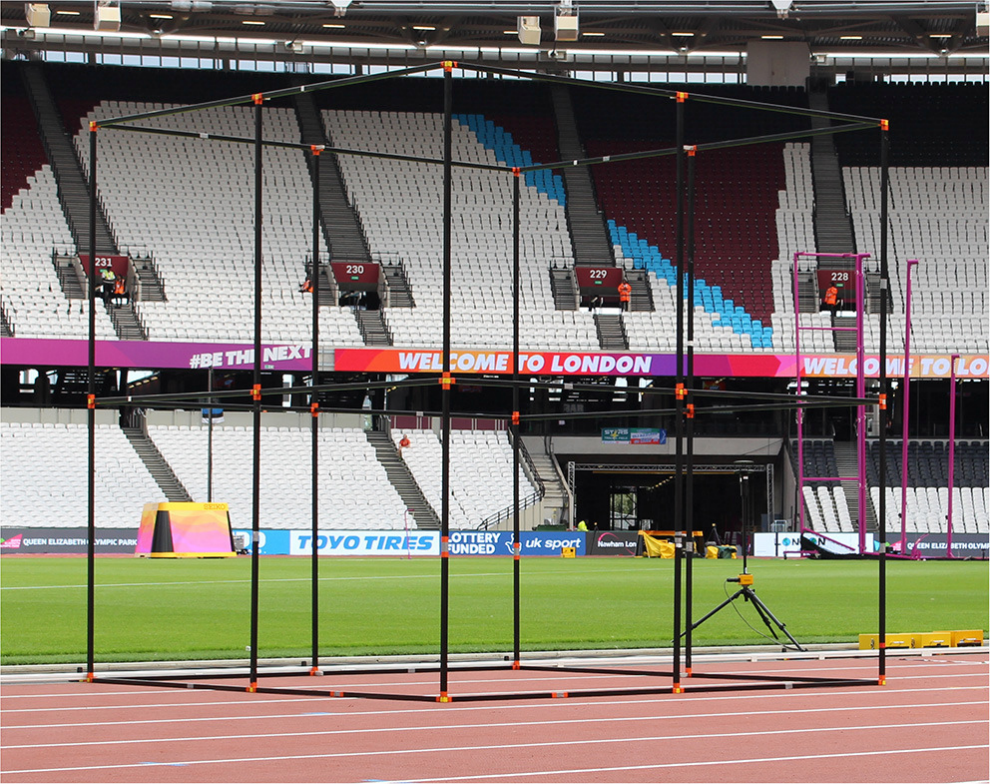
Calibration frame used in the current study (3.044 m^3^). The calibration frame pictured was placed at various locations across and along the running track, ensuring the full hurdle clearance was calibrated for each athlete.

All data were collected using four Sony PXW-FS7 high-speed cameras (150 Hz; shutter speed: 1/1250 s; ISO: 2000-4000; FHD: 1920 × 1080 px). Cameras were stationary and positioned along the home straight to focus on the sixth and fifth hurdle for the men's and women's event, respectively. These hurdles were analyzed because the allocated camera positions necessitated the analysis of the mid-section of the track (a hurdle position with 50.58 and 53.00 m remaining for men and women, respectively). A calibration procedure was carried out before and after each event using a rigid cuboid calibration frame (3.044 m^3^) that comprised 24 control points (Figure 1). The frame was positioned in six specific, predefined locations along and across the track to ensure an accurate definition of a volume covering the area around the hurdle for all eight lanes. This approach produced a large number of non-coplanar control points per calibrated volume and facilitated the construction of bi-lane local coordinate systems, which were then combined into a global coordinate system.

### Data Analysis

**Figure 2:**
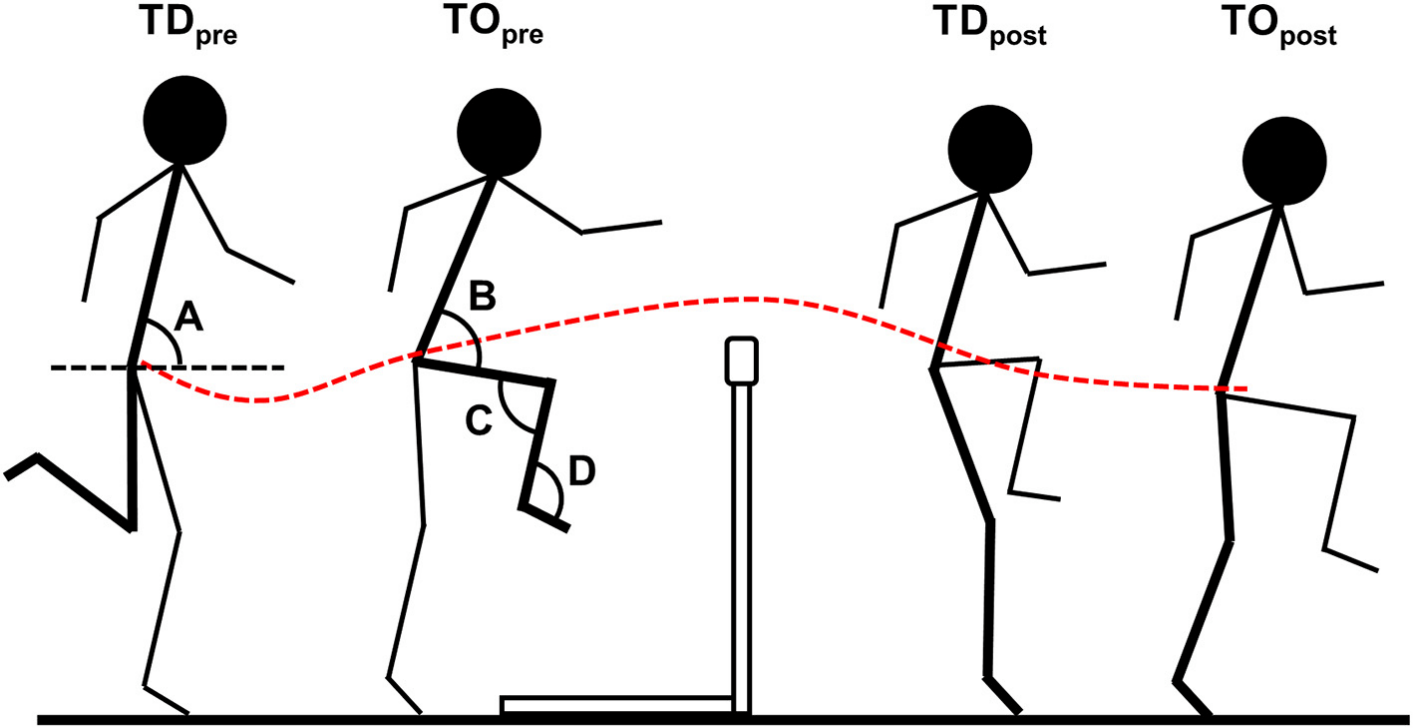
Representative schematic of the four critical instants used in the current study (TD_pre_, TO_pre_, TD_post_, and TO_post_). Bold segments represent segments analyzed. Angles analyzed are represented with A (trunk angle), B (hip angle), C (knee angle), and D (ankle angle). Red, dashed line represents center of mass path.

Video files were imported into SIMI Motion (version 9.2.2, Simi Reality Motion Systems GmbH, Germany) and manually digitized by a single experienced operator to obtain whole-body kinematic data. To synchronize the two-dimensional coordinates from each camera, an event synchronization technique (synchronization of four key instants: touchdown before hurdle clearance [TD_pre_], take-off before hurdle clearance [TO_pre_], touchdown after hurdle clearance [TD_post_], and toe-off after hurdle clearance [TO_post_]) was applied (Figure 2). Each athlete file was then digitized frame-by-frame and, upon completion, adjustments were made using the points-over-frame method (Bahamonde and Stevens, 2006). A whole-body approach was taken with the digitizing process using a model consisting of 17 points: the center of the head, and the left and right shoulder, elbow, wrist, metacarpo-phalangeal, hip, knee, ankle, and metatarso-phalangeal joint centers in accordance with de Leva (1996). The continuous digitizing process covered each frame from TD_pre_ to TO_post_, plus 10 preceding and 10 succeeding frames that acted as a buffer during filtering. The Direct Linear Transformation algorithm (Abdel-Aziz et al., 2015) was used to reconstruct the three-dimensional coordinates from individual camera's x- and y-image coordinates. The reliability of this digitizing process has been described in previous studies using the same methodological approach (including same operator) (Bezodis et al., 2019; Bissas et al., 2022). In addition, as time-series data are being compared here, one joint angle (lead leg knee angle) for a randomly selected athlete was digitized throughout the entire analyzed phase on two occasions separated by a 48-h interval. The coefficient of multiple determination (CMD) between these two data arrays was computed using the MATLAB (version R2021b, MathWorks, Inc., Natick, MA) in-built function “fitlm,” and provides an indication of the waveform similarity across the length of the sample. CMD has been used previously to determine the reliability of kinematic data (Kavanagh et al., 2006) and values can range from zero to one, which indicate more different and more similar waveforms, respectively. In the current analysis CMD was 0.9865, indicating excellent waveform similarity between the two samples.

All subsequent analyses were conducted in MATLAB. The trunk segment was defined as the straight line between two virtual “central shoulder” and “central hip” markers, which were computed using the midpoint between left and right joint centers:


(1) $$\begin{array}{r} M=\left(x_{l}+\left(\frac{x_{r}-x_{l}}{2}\right),y_{l}+\left(\frac{y_{r}-y_{l}}{2}\right),z_{l}+\left(\frac{z_{r}-z_{l}}{2}\right)\;\right), \end{array}$$

where *M* represents the three-dimensional coordinates of the virtual midpoint (shoulder or hip), and *l* and *r* indicate left and right joint centers, respectively. For subsequent analyses, the three-dimensional coordinates (digitized or computed) were projected onto a two-dimensional sagittal plane using only anteroposterior and vertical coordinates. Trunk angle (A) was defined as the angle of the trunk relative to the horizontal, lead leg hip angle (B) was defined as the angle between the trunk and the thigh segments, lead leg knee angle (C) was defined as the ankle between the thigh and shank segments, and lead leg ankle angle (D) was defined as the angle between the shank and foot segments (Figure 2). CM positional data were estimated using the de Leva (1996) body segment parameter model. From these data, CM height (above the running surface) and anteroposterior CM velocity (rate of change in position) were computed. CM height was calculated as a percentage of hurdle height for each respective event (Hanley et al., 2021), and anteroposterior CM velocity was calculated as a percentage of “entry velocity” (i.e., the value at TD_pre_) to provide an indication of velocity gain or loss between different athletes and events. Finally, the CM angle of projection was also computed using the CM's x- and y-coordinate displacements (the angle calculated between the change in x- and change in y-coordinates). Variables were filtered using a low-pass, second-order recursive Butterworth filter at individualized cut-off frequencies, which were determined using residual analyses (Winter, 2009). All variables were normalized to a percentage of total time (from TD_pre_ to TO_post_) to permit groupings of time-series data and comparisons between groups.

### Statistics

All statistical analyses were conducted in MATLAB. Descriptive statistics and discrete data were all computed as means ± standard deviations. Any discrete data comparisons between men and women were conducted using an independent *t*-test with an α-level of 0.05. To compare men's and women's time-series curves, SPM analyses (Pataky, 2010; Pataky et al., 2013) were performed for the different variables. SPM independent *t*-tests were carried out using the freely available “spm1d” software package (version M.0.4.7). A significant suprathreshold cluster was determined when the SPM *t*-statistic exceeded the upper or lower threshold corresponding with α = 0.05.

## Results

The men completed the final in 13.27 ± 0.12 s, 3% slower than the world leading time when the event took place (12.90 s). The women completed the final in 12.76 ± 0.14 s, 4% slower than the world leading time when the event took place (12.28 s). The time between TD_pre_ and TO_post_ was 0.550 ± 0.012 s for the men, which was longer than the 0.494 ± 0.014 s taken by the women (*t* = 8.63, *p* < 0.001). Although no athletes knocked over the hurdle being analyzed, three men and three women made light-moderate contact with the hurdle during clearance (Pollitt et al., 2018a,b).

Discrete joint kinematics for the men and women at key events during the hurdle phase are presented in Table 1. Men had a higher trunk angle at TD_pre_ (*p* = 0.018), but there were no differences at other key events of the hurdle phase (*p* ≥ 0.173). Men also had a lower (more flexed) hip angle in the lead leg at TO_pre_ (*p* = 0.029), although this was not different at TD_pre_ or after hurdle clearance (*p* ≥ 0.224). There were no differences between men and women in knee angle before hurdle clearance (*p* ≥ 0.199), but men had a more extended knee at TD_post_ (*p* = 0.037) and a more flexed knee at TO_post_ (*p* = 0.003), which also produced a greater flexion range for men (*p* = 0.001). There were no differences in ankle angle between men and women (*p* ≥ 0.317).

**Table 1:** Kinematic characteristics of men and women at key events (TD_pre_, TO_pre_, TD_post_, TO_post_).

		**Men**	**Women**	***t*-value**	***p*-value**	**Difference**
Trunk angle (°)	TD_pre_	84 ± 3	80 ± 3	2.69^*^	0.018	*M > W*
	TO_pre_	73 ± 6	72 ± 2	0.50	0.624	
	TD_post_	61 ± 6	59 ± 6	0.51	0.619	
	TO_post_	77 ± 5	74 ± 4	1.44	0.173	
Hip angle (°)	TD_pre_	172 ± 2	171 ± 3	0.60	0.555	
	TO_pre_	68 ± 10	78 ± 6	−2.43^*^	0.029	*M < W*
	TD_post_	60 ± 4	57 ± 5	1.27	0.224	
	TO_post_	167 ± 8	168 ± 5	−0.38	0.713	
Knee angle (°)	TD_pre_	79 ± 10	75 ± 10	0.85	0.410	
	TO_pre_	74 ± 3	69 ± 11	1.35	0.199	
	TD_post_	166 ± 10	156 ± 9	2.31^*^	0.037	*M > W*
	TO_post_	138 ± 10	151 ± 3	−3.59^**^	0.003	*M < W*
	Dif. TD-TO_post_	29 ± 14	5 ± 9	3.98^**^	0.001	*M > W*
Ankle angle (°)	TD_pre_	139 ± 7	142 ± 5	−0.88	0.395	
	TO_pre_	109 ± 10	103 ± 11	1.04	0.317	
	TD_post_	130 ± 11	126 ± 7	0.94	0.362	
	TO_post_	151 ± 6	150 ± 3	0.23	0.823	

* *Denotes significant difference at p < 0.05*;

** *Denotes significant difference at p < 0.01*.

**Figure 3:**
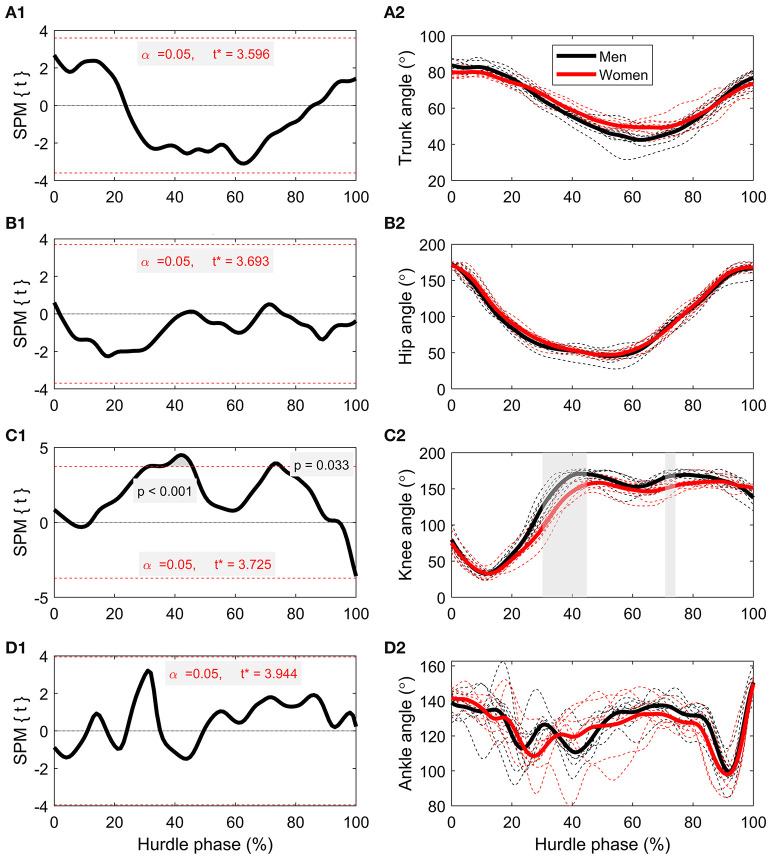
**(A1,A2)** Time-series comparisons between men and women for trunk angle, **(B1,B2)** hip angle, **(C1,C2)** knee angle, and **(D1,D2)** ankle angle. The left-hand column contains the SPM *t*-statistics curves, and the right-hand column contains grouped mean curves (thicker, solid lines) as well as individual athlete data (thinner, dashed lines) for men (black lines) and women (red lines). Gray-shaded regions correspond with suprathreshold clusters detected through SPM.

There were no differences between men's and women's time-series trunk angle (Figures 3A1,A2) or hip angle (Figures 3B1,B2). Suprathreshold clusters were detected for knee angle between 30 and 45% (*p* < 0.001), and 71–74% (*p* = 0.033) of the hurdle phase, with men showing a more extended knee angle during hurdle clearance (Figures 3C1,C2). There were no differences between men and women for ankle angle (Figures 3D1,D2).

**Figure 4:**
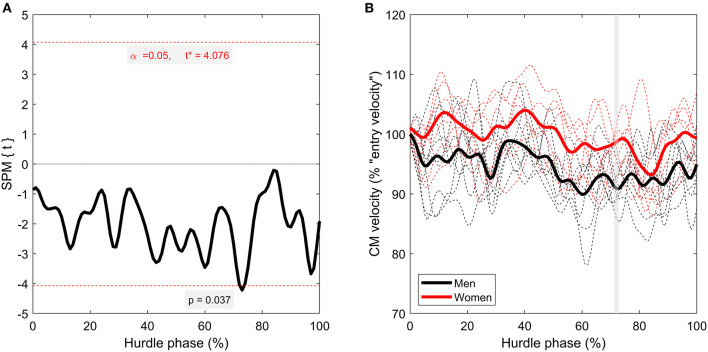
Time-series comparison between men and women for CM anteroposterior velocity (computed as a percentage of “entry velocity” [i.e., velocity at TD_pre_]). The left-hand figure **(A)** contains the SPM *t*-statistic curve, and the right-hand figure **(B)** contains grouped mean curves (thicker, solid lines) as well as individual athlete data (thinner, dashed lines) for men (black lines) and women (red lines). Gray-shaded regions correspond with suprathreshold clusters detected through SPM.

**Figure 5:**
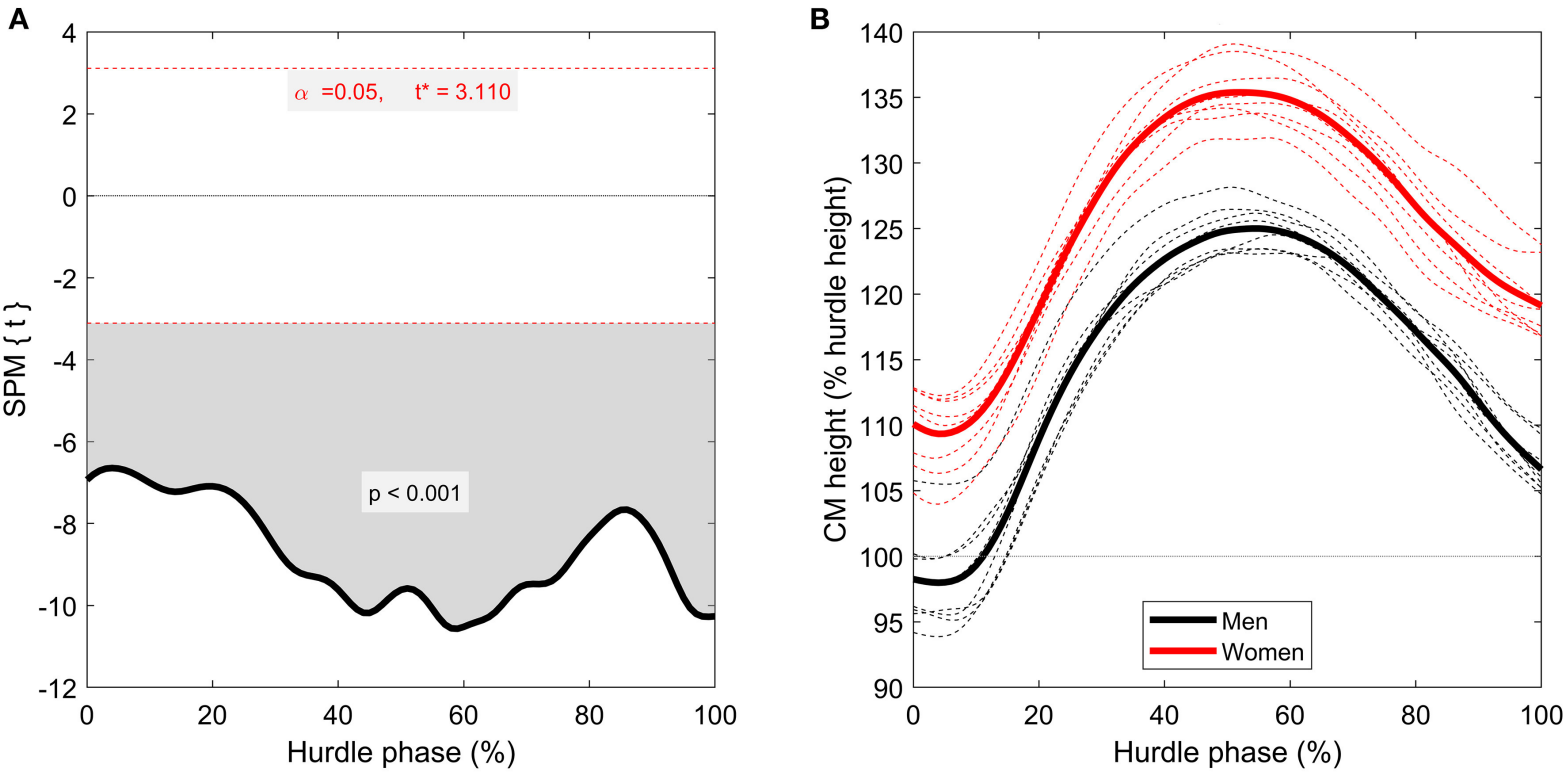
Time-series comparison between men and women for CM height (computed as a percentage of hurdle height). The left-hand figure **(A)** contains the SPM *t*-statistic curve, and the right-hand figure **(B)** contains grouped mean curves (thicker, solid lines) as well as individual athlete data (thinner, dashed lines) for men (black lines) and women (red lines). There is no gray-shaded region on this occasion, as the whole time-series curve exceeded the critical *t*-statistic threshold.

**Figure 6:**
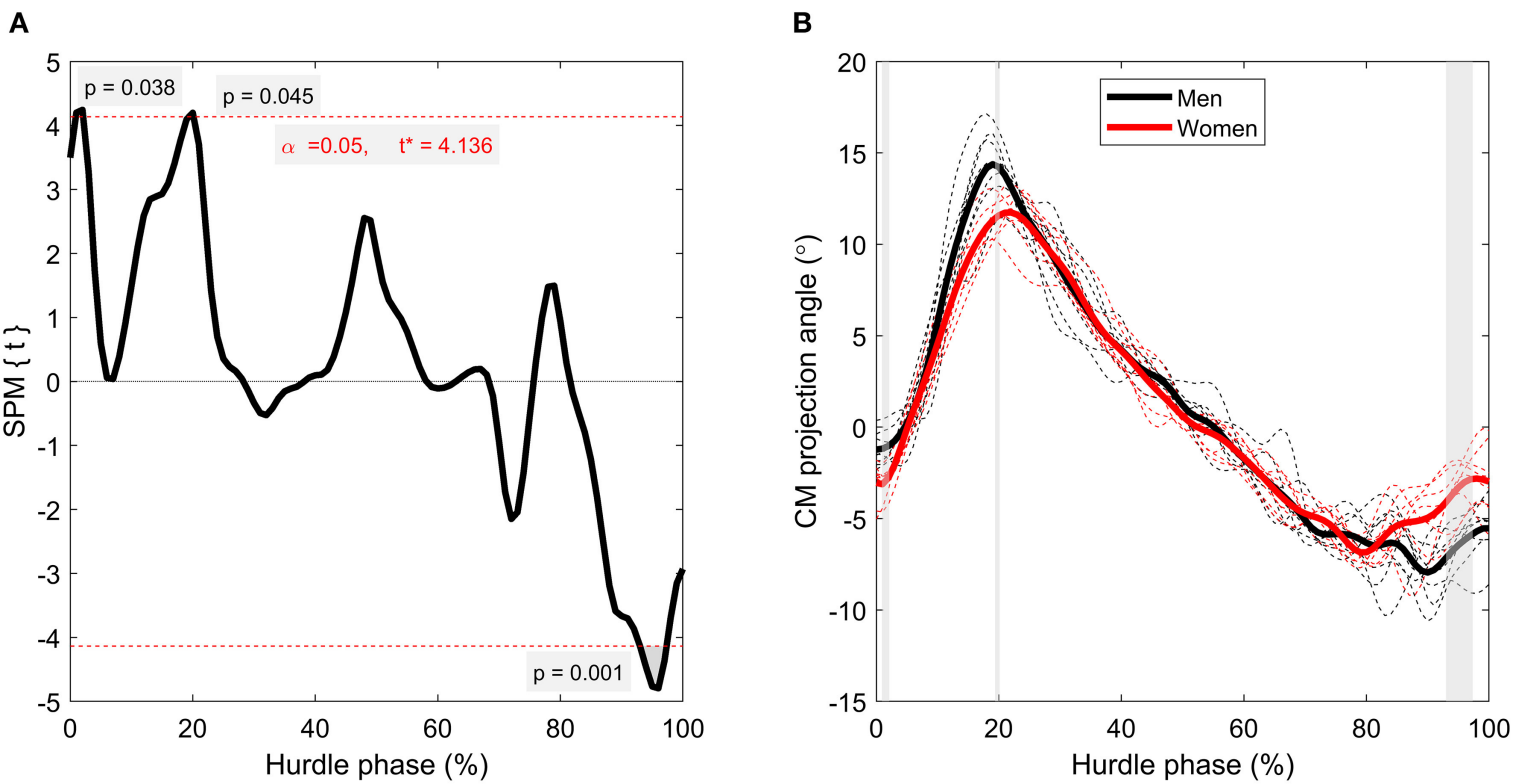
Time-series comparison between men and women for CM angle of projection. The left-hand figure **(A)** contains the SPM *t*-statistic curve, and the right-hand figure **(B)** contains grouped mean curves (thicker, solid lines) as well as individual athlete data (thinner, dashed lines) for men (black lines) and women (red lines). Gray-shaded regions correspond with suprathreshold clusters detected through SPM.

SPM analysis detected short yet significant differences between men and women around 71–73% (*p* = 0.037) of the hurdle phase for CM velocity (Figure 4), where women were faster than men. Although only statistically significant in that region, women had higher relative CM velocity throughout the entire time-series, even exceeding “entry velocity” in some instances. CM height, as a percentage of hurdle height, was greater in the women throughout the entire hurdle phase (*p* < 0.001; Figure 5), with almost all women displaying higher values than all men throughout the analyzed phase (minimal crossover). There were also brief suprathreshold clusters detected for CM projection angle, with men displaying a higher angle around 1–2% (*p* = 0.038) and 19–20% (*p* = 0.045) of the hurdle phase. In addition, men had a lower CM projection angle around TO_post_ (93–97%; *p* = 0.001) compared with the women (Figure 6).

## Discussion

The overall aim of this study was to compare joint and CM kinematic characteristics in men and women world-class hurdlers during a World Championships final. This was the first study to present time-series kinematic data around hurdle clearance and given the technical ability of the athletes analyzed, it can be used as a template when analyzing the technique of other athletes in similar competitions and training. Analyses showed that there were key kinematic differences between men's and women's hurdling techniques, especially in the knee joint of the lead leg around and after hurdle clearance. Men had to extend their knee more to clear the hurdle cleanly and, in turn, landed with a more extended knee, which then flexed more throughout the landing step. CM height, when displayed relative to hurdle height, was also different between groups, with women having a greater CM height than men throughout the entire hurdle clearance phase. Furthermore, there were few statistically significant discrepancies in CM velocity loss between groups, but it seemed that women were more efficient by maintaining and even exceeding their entry velocity for the first 40% of the hurdle phase which corresponds to the take-off phase (TD_pre_ to TO_pre_) and some of the flight phase.

An important and still contemporary concept for debate in senior hurdling is the height of the women's hurdle (Etcheverry, 1993; Stein, 2000). The current study showed that, when CM height is computed relative to hurdle height, women's values are higher than men throughout the entire hurdle clearance phase (from TD_pre_ to TO_post_; Figure 5, *p* < 0.001). This indicates that the demands of clearing the hurdle are different between women and men, with women having a larger margin of error during clearance. This provides additional evidence to our previous work (Hanley et al., 2021) that showed women are potentially at a technical advantage over the men because of the relatively lower hurdle heights they must overcome. As such, the present evidence supports the notion that an increase in the hurdle height for the women's events should be considered to match the improvements in performance over the last five decades. The technical advantage offered also meant that women were able to complete hurdle clearance (from TD_pre_ to TO_post_) in less time than men (~11% difference, *p* < 0.001) which, over the course of 10 hurdles, would result in a 0.56 s time saving (the present time difference between men's 110 m hurdles and women's 100 m hurdles is 0.60 s). However, it should be noted that although the athletes in the current study do represent the best athletes in the sport, this time saving might not be reflective of competitors below world-class competition, and further research should investigate this. With this additional information, arguments could be made to adjust hurdle heights to create a parity in hurdle clearance time, which would negate the technical and mechanical advantage currently evident.

In addition to hurdle clearance time and CM height, there were also differences in joint kinematics, both in the discrete analysis of key time points (Table 1) and in the time-series curves through SPM (Figure 3). At TO_pre_, men had a more flexed lead hip angle (*p* = 0.029), which could have been caused by the need to raise their lower limbs higher (into a more “tucked” position) to navigate the hurdle without touching it. Despite this difference, there were no suprathreshold clusters detected in SPM analyses for hip angle (Figures 3B1,B2, subplots), which can be explained by two reasons. First, the normalized time-series curves used for SPM analyses are computed from TD_pre_ to TO_post_, meaning the absolute time duration of each curve is individual-specific. As such, TO_pre_ occurred at different percentages of total hurdle time for different athletes, meaning specific time points within the normalized curves cannot be directly compared to the discrete time points presented in Table 1 (apart from TD_pre_ and TO_post_). In addition, SPM analyses maintain tighter controls over Type I and Type II statistical errors (Robinson et al., 2015), and therefore it is plausible that the difference found in trunk angle at TD_pre_ is subject to Type I error. SPM analyses did reveal between-group effects for knee angle (*p* ≤ 0.033), with suprathreshold clusters occurring around areas of maximal knee extension, showing that men extended their knee joint more than women (Figures 3C1,C2, subplots). As with the finding for hip angle at TO_pre_, this is likely because of the elevated mechanical demand of hurdle clearance; the more extended knee reduces the chance of the heel and posterior aspect of the thigh contacting the hurdle in the upward and downward phases of flight, respectively. These differences, along with the suprathreshold clusters detected for CM projection angle (Figure 6), clearly show different hurdle clearance kinematic techniques between men and women. It should be noted that it does seem that the men's response to the higher mechanical demand is somewhat successful, as an equal number of men and women made some contact with hurdle (*n* = 3), and none knocked the hurdle over completely (Pollitt et al., 2018a,b).

In addition to, or perhaps because of, the knee angle differences during flight, men also had a more extended knee at TD_post_ than women (Table 1, *p* = 0.037). As previously discussed by Hanley et al. (2021), a more extended knee at TD_post_ explains the greater CM height (when displayed relative to athlete stature) in men, even though CM height is higher in women when displayed relative to hurdle height (Figure 5). Recent work by Nagahara et al. (2021) showed that sub-elite male hurdlers must withstand >20 N/kg of vertical and >10 N/kg of anteroposterior ground reaction forces during the braking phase of the step after hurdle clearance, meaning lower body muscle-tendon complexes such as the quadriceps femoris must possess the capability to absorb very high forces upon landing. Based on the current results, this is more important for men, and training such muscle groups is required, as the same knee angle at TO_post_ was more flexed in men than women (Table 1, *p* = 0.003). Unlike flat sprinting where following TD both men and women encounter a knee depression of ~17° (Bissas et al., 2018a,b, 2022), before their knee returns to TD levels (~154°) to support the take-off of the whole body, in hurdling as our data indicates, the depression during landing following the hurdle clearance is not reversible. As a result of this, the knee angle at TO following landing corresponds for most athletes to the point of maximum knee flexion. This is logical given the powerful landing after hurdle clearance, but our findings emphasize the greater knee depression rates for men (Table 1) whilst this depression seems more attenuated in women, with three women from our sample managing a small overcompensation at TO. Therefore, it appears that men's knee joints show a “buckling” effect due to the high vertical velocity, and thus ground reaction forces, at landing. However, whether this is an effect of the relative higher hurdle height, or whether it is simply a tactical decision by the men to lower their CM in a controlled way requires further investigation.

Despite the observed kinematic differences between men and women, seemingly imposed by the discrepancy in mechanical demand caused by the different hurdle heights, no statistically significant differences were found in CM velocity loss (when presented relative to “entry velocity”) besides a short suprathreshold cluster between 71 and 73% (Figure 4). Given women had a shorter absolute hurdle time, similar velocity loss between groups could imply women had a higher entry velocity. However, the men covered a greater distance between TD_pre_ and TO_post_ (Hanley et al., 2021), meaning this was not the case. However, despite a lack of statistical significance, it was observed that women were better able to maintain velocity during the hurdle phase (Figure 5). They were even able to increase relative CM velocity at various points during the first two quarters of the hurdle phase, which the men were unable to do. The inevitably small sample size of the populations studied probably contributed a lack of statistical power here, but the observational differences are noteworthy. Therefore, the clear technical and kinematic differences between men's and women's hurdle clearance strategies might lead to different amounts of velocity loss (women appear more efficient at maintaining velocity), which must be recovered in the subsequent steps before the next hurdle.

The current study was not without some limitations. The hurdle analyzed for the men was hurdle six out of ten, whereas for the women was hurdle five out of ten. Therefore, it is possible that the two groups were at different stages of the race and therefore at different points on their velocity-time curves. The main reason for this limitation was logistical; camera positions allocated to the research team by the event organizers meant that only certain sections of the home straight could be analyzed optimally and, given hurdle six for men and hurdle five for women are positioned at similar positions with respect to the finish line, these were the best hurdles for analysis. However, the times between hurdles around this stage of the race are typically very similar (see Pollitt et al., 2018a,b), implying both men and women were traveling at maximum velocity through their respective capture volumes. In addition, the analyzed hurdle phase in the current study incorporated both contact and flight phases, which could mean parts of our normalized curves represent different parts of the movement for men and women. However, contact phases both before and after the hurdle are important components of hurdle clearance, so should be included as part of this normalized curve. Our analysis focused on the lower body with a partial representation of upper body kinematics (trunk angle). The upper body including the movements of the arms affect the trajectory of the center mass over the hurdle and therefore play a part in the effectiveness of hurdle clearance. Since there is a large portion of arm movement in the frontal and transverse planes during hurdling clearance and our methodological approach was (although appropriate for 3D motion capture) optimized for capturing sagittal plane motion, we limited our analysis to sagittal kinematics. The role of the upper limbs is important and should be considered in future lab-based studies with access to optoelectronic motion capture systems and comprehensive marker sets. A further limitation was that the sample was limited to eight athletes in each race, but this set up delivered high ecological validity as the data are of world-class hurdlers competing in World Championship finals. Nonetheless, the performance standard of the athletes in this study means that data can be used for “gold-standard” performance models but might not be applicable to larger populations (elite, highly trained) of sprint hurdlers. Further research should seek to replicate similar studies at track and field competitions with lower entry standards (national/continental championships or even regional events) to develop an understanding of how these characteristics change across performance abilities.

Finally, because of the location of data collection, our methodological approach was, naturally, restricted to high-speed videography only. Other biomechanical measurements such as ground reaction forces or of muscle activity would provide more information about the mechanical demand of hurdling but are not possible in a field-based setting during competition on a regulated running surface. Despite this, the kinematic and temporal data presented in the current study provide a novel insight into the performance of world-class hurdling, which future studies might be able to build on in a laboratory setting, where other biomechanical measurements are possible.

## Conclusions

In summary, this was the first study to present time-series joint kinematic CM positional and velocity data for world-class hurdlers during a World Championships final. Comparisons between men and women revealed women to have a greater CM height (relative to hurdle height) throughout the entire hurdle clearance phase and were better to maintain their horizontal velocity during the hurdle phase. The discrepancy in hurdle heights also caused men to have a more flexed hip joint at TO_pre_, a more extended knee joint at intervals throughout the flight phase and at landing, which also caused greater knee flexion at TO_post_. The men's knee joints during the first impact after the hurdle clearance showed a “buckling” effect due to the high vertical velocity, and thus ground reaction forces, at landing. The hurdling conditions also meant that women were able to complete hurdle clearance in less time than men. The study confirms and expands our recent research findings and supports the argument that women are at a technical and mechanical advantage over men because of their relatively lower hurdle heights in their event, which could be reviewed by governing bodies.

## Data Availability Statement

The datasets generated for this study will not be made available in order to avoid identifying individual athletes. Requests to access the datasets should be directed to AB, a.bissas@athleticsbiomechanics.com.

## Ethics Statement

The studies involving human participants were reviewed and approved by Carnegie School of Sport Research Ethics Committee, Leeds Beckett University. The patients/participants provided their written informed consent to participate in this study.

## Author Contributions

AB and SM arranged data collection during the World Championships as Project Director and Project Leader, respectively. AB, JW, and BH performed data collection. JW, AB, and GP processed the data. JW created the figures. All authors interpreted the results of the research, conceptualized, designed the study, wrote the manuscript, edited, critically revised, and approved the final version for submission.

## Funding

The data collection and initial data analysis were supported by funding provided by the IAAF/World Athletics as part of a wider Development/Education project; however, the nature of the data is purely descriptive and not associated with any governing body, commercial sector, or product. The results of the present study do not constitute endorsement by the World Athletics.

## Conflict of Interest

The authors declare that the research was conducted in the absence of any commercial or financial relationships that could be construed as a potential conflict of interest.

## Publisher's Note

All claims expressed in this article are solely those of the authors and do not necessarily represent those of their affiliated organizations, or those of the publisher, the editors and the reviewers. Any product that may be evaluated in this article, or claim that may be made by its manufacturer, is not guaranteed or endorsed by the publisher.
